# Performance of the international 2023 myelin oligodendrocyte glycoprotein antibody-associated disease panel criteria in a Japanese cohort: combined assessment of myelin oligodendrocyte glycoprotein immunoglobulin G in serum and CSF

**DOI:** 10.1093/braincomms/fcag420

**Published:** 2026-11-02

**Authors:** Yuki Matsumoto, Naoki Yamamoto, Kimihiko Kaneko, Yoshiki Takai, Toshiyuki Takahashi, Tatsuro Misu, Kazuo Fujihara, Masashi Aoki

**Affiliations:** Department of Neurology, Tohoku University Graduate School of Medicine, Sendai 980-8574, Japan; Department of Neurology, Tohoku University Graduate School of Medicine, Sendai 980-8574, Japan; Department of Neurology, Tohoku University Graduate School of Medicine, Sendai 980-8574, Japan; Department of Neurology, Tohoku University Graduate School of Medicine, Sendai 980-8574, Japan; Department of Neurology, National Hospital Organization Yonezawa National Hospital, Yonezawa 992-1202, Japan; Department of Neurology, Tohoku University Graduate School of Medicine, Sendai 980-8574, Japan; Department of Multiple Sclerosis Therapeutics, Fukushima Medical University, Fukushima 960-1295, Japan; Multiple Sclerosis & Neuromyelitis Optica Center, Southern TOHOKU Research Institute for Neuroscience, Koriyama 963-8563, Japan; Department of Neurology, Tohoku University Graduate School of Medicine, Sendai 980-8574, Japan

**Keywords:** myelin oligodendrocyte glycoprotein IgG, MOG-antibody-associated disease, diagnostic criteria, aseptic meningitis

## Abstract

In the 2023 International Panel's diagnostic criteria for myelin oligodendrocyte glycoprotein antibody-associated disease (MOGAD), one of six core clinical demyelinating events (core events) is required: optic neuritis (ON), myelitis, acute disseminated encephalomyelitis (ADEM), cerebral monofocal or polyfocal deficits, brainstem or cerebellar deficits, and cerebral cortical encephalitis. If myelin oligodendrocyte glycoprotein immunoglobulin G (MOG-IgG) in serum is not clearly positive or is CSF restricted, the criteria require supporting clinical and MRI features (supporting features). We assessed the performance of the MOGAD panel criteria in relation to MOG-IgG titres in serum and CSF, and explored the diagnostic implications of CSF MOG-IgG as well as atypical clinical presentations.

In this cross-sectional cohort study, we analysed 405 cases of non-multiple-sclerosis CNS demyelinating diseases with aquaporin 4 immunoglobulin G negativity, all of whom underwent MOG-IgG testing using paired serum and CSF samples collected across Japan from 2015 to 2018. Endpoint titres were determined by two-fold serial dilutions (starting at 1:16 for serum and 1:1 for CSF), with ≥1:128 in serum defined as the cut-off to minimize false-positive results, as we previously established. Two independent investigators evaluated whether each patient met the core events and the supporting features.

A total of 141 cases exhibited MOG-IgG positivity in serum and/or CSF. The core events included ON (*n* = 50, 36%), myelitis (*n* = 61, 43%), ADEM (*n* = 31, 22%), cerebral deficits (*n* = 22, 16%), brainstem/cerebellar deficits (*n* = 26, 18%) and cortical encephalitis (*n* = 22, 16%) with overlap. Five cases (3.5%) did not meet the criteria; two cases of meningitis with headache and prolonged fever lacked core events, while one case of ON and two cases of myelitis lacked the supporting features and showed either low-positive serum or CSF-restricted MOG-IgG. The rate of meeting the supporting features showed a titre-dependent trend; 1:16–1:64, 88%; 1:128–1:512, 97%; ≥1:1024, 100% in serum; negative–1:4, 92%; 1:8–1:32, 100%; ≥1:64, 100% in CSF.

Overall, the criteria demonstrated good performance. In our cohort, all cases with high CSF positivity (≥1:8) fulfilled the supporting features. Future revisions may need to incorporate less common presentations, such as meningitis or prolonged fever with clear positivity and the supporting features, and recognize high CSF MOG-IgG titres as diagnostic. Additionally, in some cases with low positivity or CSF-restricted MOG-IgG that do not meet the supporting features, MOGAD may remain a possible diagnosis after careful exclusion of alternative diagnoses.

## Teaser

Matsumoto *et al*. report that the 2023 diagnostic criteria for myelin oligodendrocyte glycoprotein antibody-associated disease performed well in a Japanese cohort with paired serum and CSF testing. High CSF antibody titres were diagnostically reliable, while meningitis-like presentations and some ON or myelitis cases were missed.

## Graphical Abstract

**Graphical Abstract:**
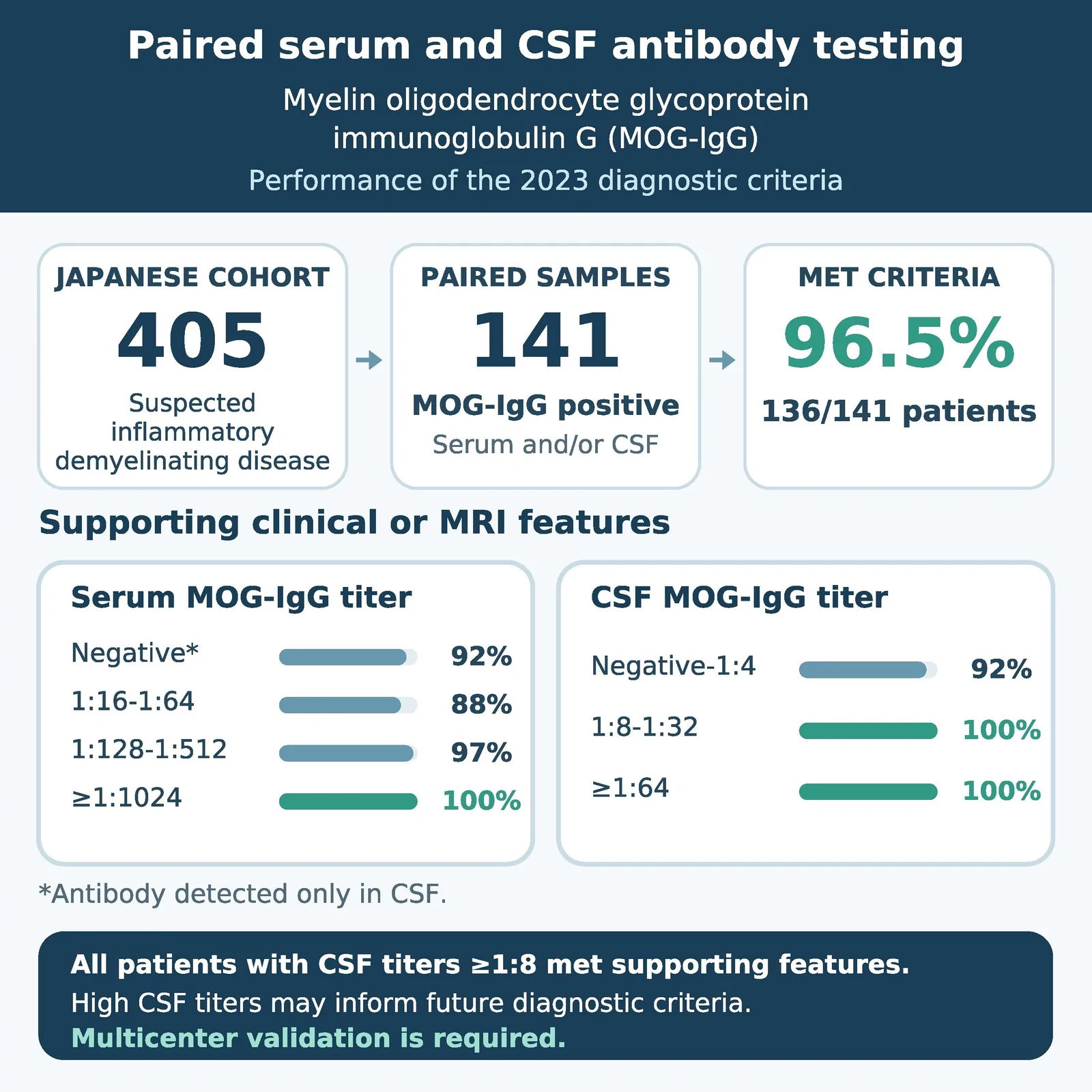

## Introduction

Myelin oligodendrocyte glycoprotein antibody-associated disease (MOGAD) is a newly recognized CNS demyelinating disorder. The international panel of MOGAD proposed new diagnostic criteria in 2023.^1^ The criteria necessitate the presence of at least one of six core clinical demyelinating events; optic neuritis (ON), myelitis, acute disseminated encephalomyelitis (ADEM), cerebral monofocal or polyfocal deficits, brainstem or cerebellar deficits, and cerebral cortical encephalitis often with seizures, along with MOG-IgG positivity and exclusion of alternative diagnoses. In cases where MOG-IgG is low-positive in serum, positive without reported titre, or CSF-restricted positive, the confirmation of aquaporin 4 (AQP4)-IgG negativity and fulfilment of one or more supporting clinical or MRI features are also required.

Several studies have demonstrated the reliability of serum-based diagnostic criteria for MOGAD, highlighting the high specificity of MOG-IgG in serum.^2-8^ However, recent studies have reported that some MOGAD cases (0–23%) showed CSF-restricted MOG-IgG, underscoring the importance of testing MOG-IgG in CSF.^9-15^ The evidence regarding the performance of the diagnostic criteria for combined assessment of MOG-IgG in serum and CSF remains limited.

The objective of this study was to evaluate the performance of the MOGAD panel criteria in relation to MOG-IgG status in serum and CSF. Previously, we reported the clinical relevance of MOG-IgG in CSF among cases of CNS demyelinating diseases with paired serum and CSF samples.^12^ However, the report was not based on the MOGAD panel criteria. Utilizing this cohort, we evaluated the criteria by assessing the frequency of the core events and the supporting features fulfilment according to the titres of MOG-IgG in serum and CSF. In addition, we examined how atypical clinical phenotypes relate to the MOGAD panel criteria and assessed the diagnostic implications of CSF MOG-IgG. We also reviewed relevant literature to contextualize these findings.

## Materials and methods

### Patients

In this retrospective cross-sectional study, we reviewed the records of 4785 consecutive patients from across Japan whose sera and/or CSF were sent to our laboratory for MOG-IgG testing between August 1, 2015 and January 31, 2018 (Fig. 1).^12^ We included patients with both serum and CSF samples obtained before any treatment in the acute phase (<1 month after onset). As previously reported, we identified 405 suspected MOGAD cases with paired serum and CSF samples before treatment, excluding those with multiple sclerosis, AQP4-IgG-positive neuromyelitis optica spectrum disorder (NMOSD) and unknown or alternative diagnoses.^12^ All patients underwent brain with/without spinal cord MRIs during the acute phase.

**Figure 1:**
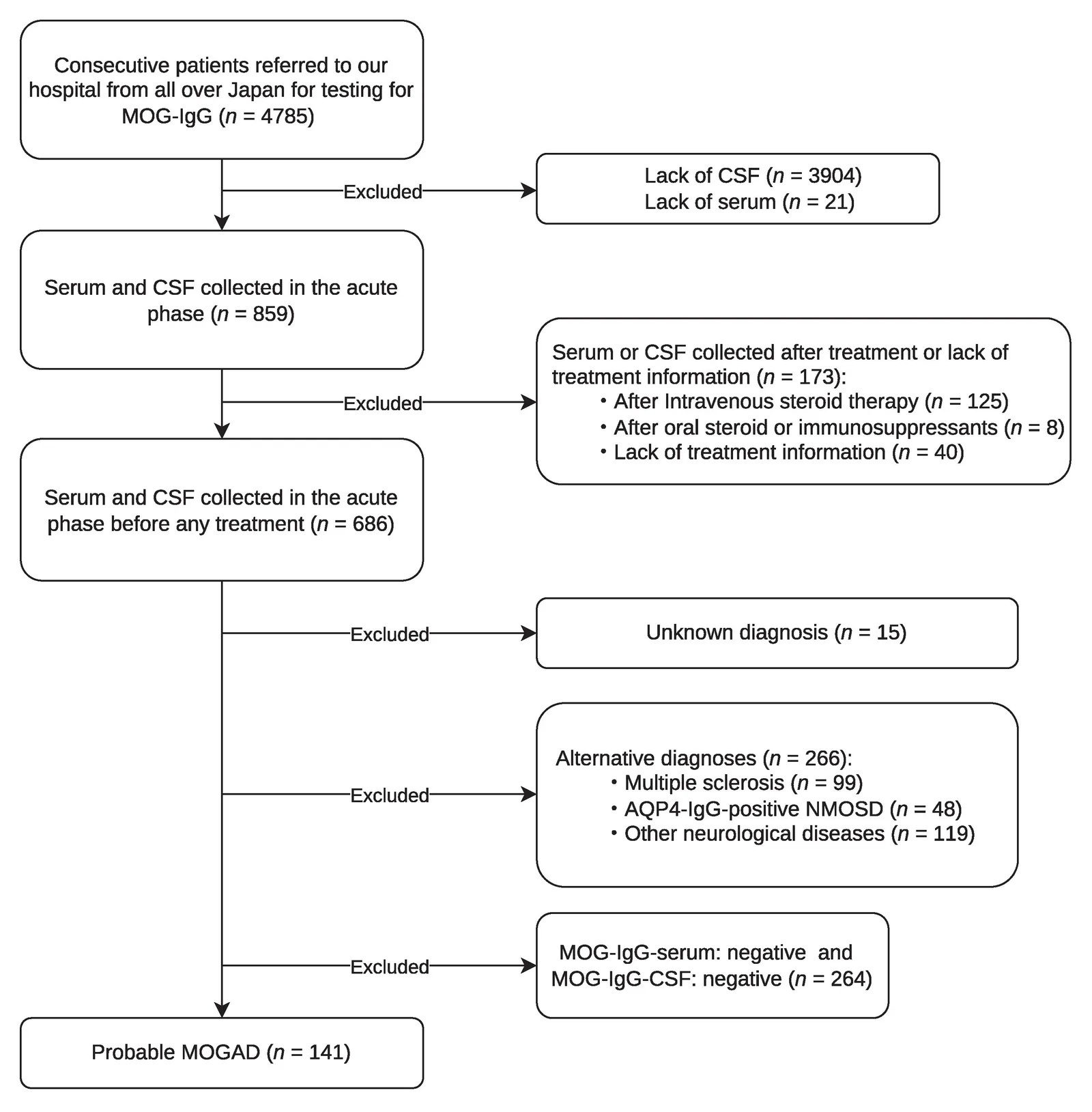
**Flowchart of patient inclusion.** A total of 4785 patients underwent MOG-IgG testing. Exclusions were made due to the absence of CSF (*n* = 3904) or serum (*n* = 21). Among 859 patients with paired serum and CSF collected in the acute phase, 173 were excluded because of prior treatment or missing treatment information. Of the remaining 686 untreated cases, 15 had an unknown diagnosis, and 266 had alternative diagnoses, including MS (*n* = 99), AQP4-IgG-positive NMOSD (*n* = 48), and other neurological diseases (*n* = 119). Additionally, 264 MOG-IgG-negative cases were excluded, resulting in 141 cases classified as probable MOGAD. AQP4, aquaporin 4; IgG, immunoglobulin G; MOG, myelin oligodendrocyte glycoprotein; MOGAD, myelin oligodendrocyte glycoprotein antibody-associated disease; MS, multiple sclerosis; NMOSD, neuromyelitis optica spectrum disorder.

### MOG-IgG assays

MOG-IgG was evaluated using an in-house live cell-based assay employing HEK293 cells expressing full-length human MOG.^12,16^ Both serum and CSF samples were tested using the same human IgG1 Fc-specific secondary antibody: mouse anti-human IgG1 Fc, clone HP6069, Alexa Fluor 488-conjugated (Invitrogen/Thermo Fisher Scientific, catalogue no. A-10631, RRID: AB_2534050). Endpoint titres were determined by serial two-fold dilutions starting from 1:16 in serum and from undiluted (1:1) CSF to sufficiently minimize false-positive results.^12^

### Clinical and MRI assessments

Two independent investigators (Y.M. and N.Y.) evaluated whether the patients met the criteria for the core events and the supporting features. Although classification based on a single dominant core event was considered, many patients presented with overlapping syndromes (e.g. concomitant ON and myelitis), making it difficult to assign a unique dominant phenotype. To avoid arbitrary classification, overlapping core events were allowed and analysed accordingly. Specifically, in patients with multiple concurrent core events, the supporting features were assessed separately for each anatomical region; for example, in cases with both ON and myelitis, the supporting features were evaluated independently for the optic nerve and the spinal cord. For the purpose of this cross-sectional validation study, the core events and the supporting features were assessed based on the attack at the time of MOG-IgG testing. Previous demyelinating events were recorded as clinical history but were not used as qualifying events in the primary criteria assessment.

For assessment of the supporting features in individual core events, cases with ON were examined for bilateral simultaneous clinical involvement, longitudinal optic nerve involvement on MRI, presence of perineural optic sheath enhancement if contrast enhanced and optic disc oedema. Similarly, cases of myelitis were analysed for longitudinally extensive lesions, central cord lesions or H-sign and conus lesions. Cases of ADEM, cerebral deficits, brainstem/cerebellar deficits and cortical encephalitis were investigated for the presence of multiple ill-defined T2 hyperintense lesions in supratentorial and often infratentorial white matter, deep grey matter involvement, ill-defined T2-hyperintensity involving pons, middle cerebellar peduncle, or medulla, and cortical lesion with or without lesional and overlying meningeal enhancement.^1^ If disagreement arose, a third investigator (K.F.) made the final decisions.

### Statistical analysis

Categorical variables were tested using a Fisher's exact test or χ^2^ test depending on the expected frequencies. Continuous variables were assessed using the Wilcoxon rank-sum test. Correlations between the supporting features and titres were assessed by Spearman's rank correlation. A two-tailed *P* value < 0.05, adjusted for multiple comparisons using the Bonferroni correction when necessary, was deemed statistically significant. All analyses were performed using RStudio (Posit, Boston, USA, Version 2024.04.2+764).

### Standard protocol approvals, registration and patient consent

The Institutional Review Board of Tohoku University Graduate School of Medicine approved this study (approval number: 2024-1-746). All study participants provided written informed consent.

## Results

### Patient characteristics

A total of 141 cases exhibited MOG-IgG titres in serum exceeding 1:16 and/or in CSF exceeding 1:1 (Table 1). The median age (interquartile range [IQR]) during this study was 26 (11–42). Forty-five percent of this cohort were female. MOG-IgG was examined at the time of relapse in 40 cases. A total of 139 cases (99%) fulfilled the core events, including 50 (36%) with ON, 61 (43%) with myelitis, 31 (22%) with ADEM, 22 (16%) with cerebral deficits, 26 (18%) with brainstem/cerebellar deficits and 22 (16%) with cortical encephalitis (duplications included), although a single case of ADEM could not be assessed for the presence of ON because of prolonged encephalopathy.

**Table 1:** Patient characteristics

Enrolled patients			*N* = 141
Age, median (IQR), y			26 (11, 42)
Female, *n* (%)			63 (45%)
Core events^a^, *n* (%)		ON	50/140^b^ (36%)
		Myelitis	61/141 (43%)
		ADEM	31/141 (22%)
		Cerebral mono/polyfocal deficits	22/141 (16%)
		Brainstem cerebellar deficits	26/141 (18%)
		Cerebral cortical encephalitis	22/141 (16%)
		None of the above	2/141 (1.4%)
Supporting features, *n*/*N* (%)^c^	ON (*N* = 50)	Bilateral simultaneous involvement	18/48 (38%)
		Longitudinal optic nerve involvement	32/46 (70%)
		Perineural optic nerve sheath enhancement	16/43 (37%)
		Optic disc oedema	23/46 (50%)
		None of the above	4/50 (8.0%)
	Myelitis (*N* = 61)	Longitudinally extensive myelitis	40/61 (66%)
		Central cord lesion or H sign	41/56 (73%)
		Conus lesion	20/59 (34%)
		None of the above	10/61 (16%)
	Cerebral, brainstem and cerebellar syndrome (*N* = 70)	Multiple ill-defined T2 hyperintense lesions in supratentorial and often infratentorial white matter	38/70 (54%)
		Deep grey matter involvement	29/70 (41%)
		Ill-defined T2-hyperintensity involving pons, middle cerebellar peduncle, or medulla	31/70 (44%)
		Cortical lesion	24/70 (34%)
		None of the above	0 (0%)
	Without any supporting features		4 (2.8%)
EDSS at nadir, median (IQR)			3.5 (2.0, 5.0)
Onset: relapse			101: 40
CSF WBC (/mm^3^), median (IQR)			17 (4, 66)
High IgG index^d^, *n*/*N* (%)			18/88 (20%)
OCB positivity, *n*/*N* (%)			8/70 (11%)
CSF MBP (pg/ml), median (IQR)			73 (20, 388)

ADEM, acute disseminated encephalomyelitis; CSF, cerebrospinal fluid; EDSS, expanded disability status scale; IgG, immunoglobulin G; IQR, interquartile range; MBP, myelin basic protein; OCB, oligoclonal band; ON, optic neuritis; WBC, white blood cell.

^a^Duplication is allowed. (e.g. cases with optic neuritis and myelitis were counted in both ON and myelitis categories).

^b^A case of ADEM could not be assessed for visual impairment due to prolonged encephalopathy.

^c^The cases without assessment were excluded from the denominator.

^d^High IgG index was defined as ≥0.75.

Two paediatric cases (1.4%) with clear serum MOG-IgG positivity did not meet the criteria due to the absence of the core events (Table 2 Demographic and Clinical Characteristics of Cases Not Fulfilling a Core Event or Any Supporting Feature). One had multifocal cerebral white matter lesions (Fig. 2A) and prolonged fever lasting 14 days, without any other neurological or systemic symptoms. The other paediatric patient had a history of ADEM 2 months earlier, which had fully resolved after IVMP. The patient was seen by a local neurologist due to prolonged fever and headache lasting over 10 days without any focal neurological deficits or encephalopathy; MRI revealed bilateral thalamic lesions (Fig. 2B).

**Figure 2:**
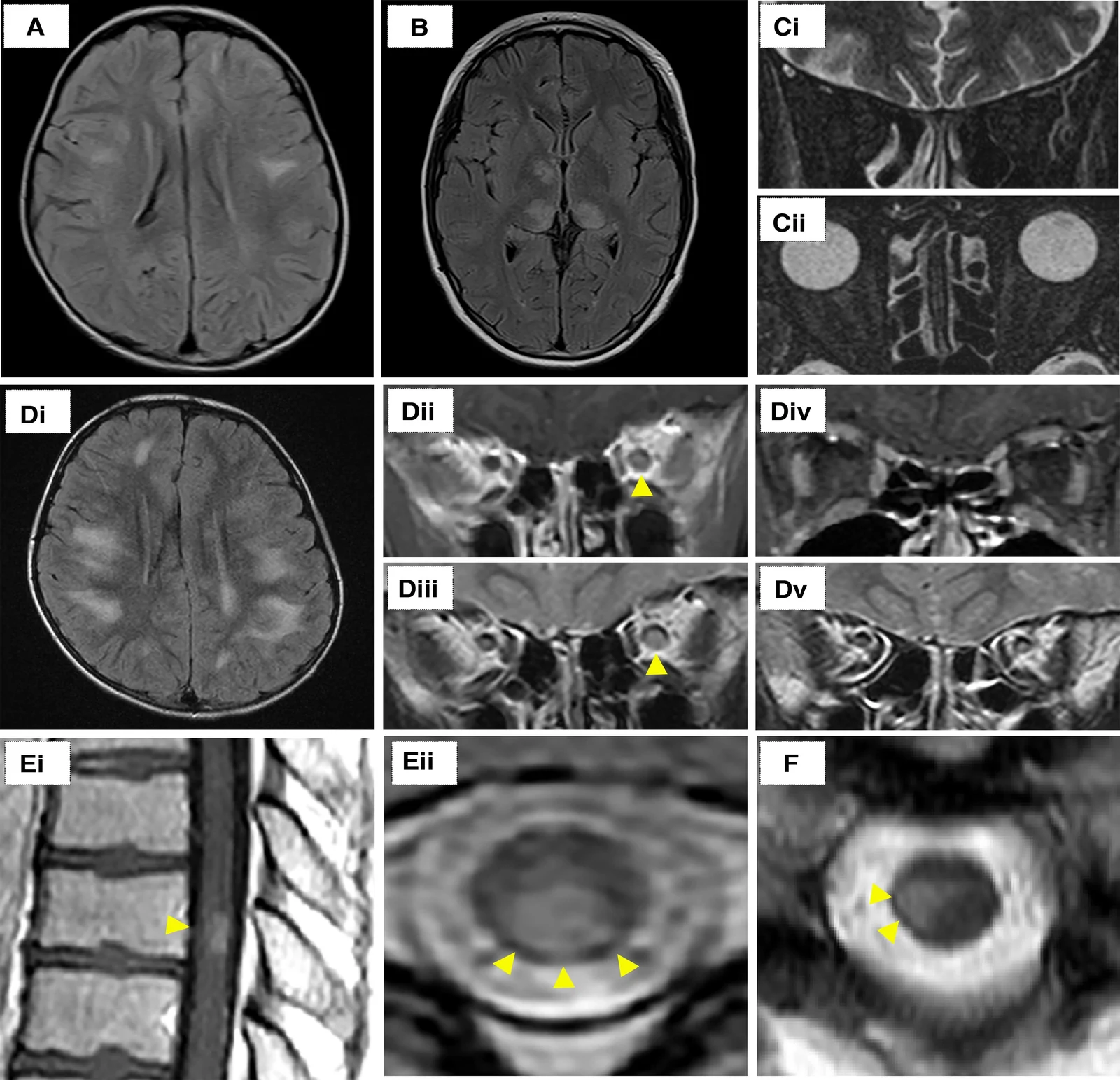
**MRI images of the cases lacking a core event or any supporting feature.** (**A**) Paediatric patient with multifocal cerebral white matter lesions and prolonged fever lasting 14 days. (**B**) Paediatric patient with a history of ADEM 2 months earlier, presenting with prolonged fever and headache; MRI showed bilateral grey matter lesions. (**C**) Orbit coronal (**Ci**) and axial (**Cii**) short tau inversion recovery MRI images of an adult male with a history of recurrent optic neuritis. MRI showed no detectable lesions. (**D**) Paediatric patient with a history of ADEM (**Di**) and left ON (**Dii, Diii**), fulfilling the supporting features; during her ON relapse, no new MRI lesions or gadolinium enhancement were observed (**Div, Dv**), and none of the supporting features were met at the time of MOG-IgG testing. (**E**) Adult patient with new-onset myelitis symptoms and a short, non-central thoracic lesion with gadolinium enhancement not fulfilling any supporting features; CSF-restricted MOG-IgG was detected. (**F**) Axial T2-weighted MRI of the cervical cord in an adult female showed a short, peripheral lesion that did not fulfil any supporting features. ADEM, acute disseminated encephalomyelitis; MOG, myelin oligodendrocyte glycoprotein; ON, optic neuritis.

**Table 2:** Demographic and clinical characteristics of cases not fulfilling a core event or any supporting feature

Age category, sex	Past history	Core events at the timing of MOG-IgG testing	The titres of MOG-IgG in serum/CSF	Supporting features	2023 IPMOGAD criteria
Paediatric, M	Unremarkable	None	1:2048 (Clear positive)/1:32	Fulfilled for multiple subcortical white matter lesions (Fig. 2A)	Not fulfilled because of lack of core events
Paediatric, M	ADEM with complete recovery by IVMP 2 months ago	None	1:2048 (Clear positive)/1:32	Fulfilled for deep grey matter lesions (Fig. 2B)	Not fulfilled because of lack of core events
Adult, M	Left ON three years ago, and right ON 2 months ago	Right ON	1:64 (Low positive)/negative	Not fulfilled because there was no new lesion of right optic nerve (Fig. 2C).	Not fulfilled because of lack of supporting features
Paediatric, F	ADEM five years ago and left ON three years ago with good recovery (both fulfilling supporting features, Fig. 2D).	Left ON	1:128 (Clear positive)/negative	Not fulfilled because there was no new lesion of left optic nerve (Fig. 2D).	Fulfilled because of ON with clear positivity of serum-MOG-IgG
Adult, M	Right ON (unclear whether supporting features criteria was met) with complete recovery 10 years ago.	Myelitis	Negative/1:2	Not fulfilled because of peripheral short cord lesion (Fig. 2E).	Not fulfilled because of myelitis with CSF-restricted MOG-IgG but not fulfilling supporting features
Adult, F	Right ON 27 years ago, Thoracic myelitis at 26 years ago and 7 years ago.	Myelitis	1:64 (Low positive)/negative	Not fulfilled because of peripheral short cord lesion (Fig. 2F)	Not fulfilled because of myelitis with low positive serum-MOG-IgG but not fulfilling supporting features.

ADEM, acute disseminated encephalomyelitis; IVMP, intravenous methylprednisolone therapy; ON, optic neuritis.

### Supporting features fulfilled in ON, myelitis and brain, brainstem or cerebellar syndrome

The agreement rate between the two independent investigators (Y.M. and N.Y.) for assessment of the supporting features was 93%, with a kappa coefficient of 0.666, indicating substantial agreement. In cases with ON (*n* = 50), bilateral simultaneous involvement was observed in 18 of 48 cases (38%), longitudinal optic nerve involvement in 32 of 46 cases (70%), perineural optic nerve sheath enhancement in 16 of 43 cases (37%) and optic disc oedema in 23 of 46 cases (50%). The number of fulfilled supporting features of ON was as follows: four cases with none (8.0%), eight cases with one (16%), 16 cases with two (32%), nine cases with three (17%), three cases with four (6.4%); records for at least one ON-related supporting feature were lacking in 11 cases (22%). The most common combination in the supporting features for ON was longitudinal involvement and disc oedema, seen in 15 cases (30%).

Among cases with myelitis (*n* = 61), the observed supporting features were as follows: longitudinally extensive myelitis occurred in 40 of 61 cases (66%), central cord lesion or H-sign was identified in 41 of 56 cases (73%, excluding five cases without axial spinal MRI images), conus lesion was found in 20 of 59 cases (34%, spinal MRI scans in two cases did not include lower thoracic cord). In 10 cases (16%), no supporting features for myelitis were fulfilled, 12 cases (20%) had one supporting feature, 22 cases (36%) had two supporting features, and 12 cases (20%) had all supporting features. In five cases (8.2%), MRI images to evaluate one or more supporting features of myelitis were unavailable. The most common combination in supporting features for myelitis was longitudinally extensive myelitis and central cord lesion or H sign, identified in 33 cases (54%).

Additionally, in cases of brain, brainstem, or cerebellar syndrome (*n* = 70), multiple ill-defined T2 hyperintense lesions in supratentorial and often infratentorial white matter were present in 38 of 70 cases (54%), deep grey matter involvement was noted in 29 of 70 cases (41%), ill-defined T2-hyperintensity involving pons, middle cerebellar peduncle, or medulla was seen in 31 of 70 cases (44%), and cerebral cortical lesions were observed in 24 of 70 cases (34%). All the 70 cases met one or more supporting features of cerebral and brainstem/cerebellar syndrome [38 cases (54%) had one supporting feature, 15 cases (21%) had two, 14 cases (20%) had three, and three cases (4.3%) had all of them]. Brain MRI images for the supporting feature assessment were available for all cases. The most frequent combination in the supporting features for cerebral and brainstem/cerebellar syndrome was multiple ill-defined T2 hyperintense lesions and deep grey matter involvement, seen in 25 cases (36%).

### MOG-IgG-positive cases that did not meet supporting features

Two of the four ON cases not fulfilling any supporting features of ON were complicated by myelitis and met the supporting features for myelitis. Among the 10 cases of myelitis not fulfilling the supporting features, eight cases were associated with ON and/or cerebral and brainstem/cerebellar syndrome, all meeting at least one supporting feature for ON and/or cerebral and brainstem/cerebellar syndrome. Eventually, the remaining two cases of ON (Cases 1 and 2) and two cases of myelitis (Cases 3 and 4) did not meet any supporting features due to the absence of new optic nerve lesion or the short, peripheral spinal cord lesions (Table 2).

Case 1: An adult male with a history of recurrent ON. He had developed left ON three years earlier and right ON two months earlier. MRI showed no detectable lesions (Fig. 2Ci and Cii). Serum MOG-IgG titre was 1:64, while CSF was negative. His symptoms improved following intravenous methylprednisolone and plasma exchange.

Case 2: A paediatric patient with a history of ADEM 5 years prior (Fig. 2Di) and left ON 3 years before (Fig. 2Dii and Diii), both of which fulfilled the supporting features. Later the patient experienced a relapse of left ON; however, no new lesions in the left atrophic optic nerve were observed on T2-weighted orbital MRI, fundoscopy was normal, and there was no gadolinium enhancement (Fig. 2Div and Dv).

Case 3: An adult patient with a history of right ON 10 years prior, without clear fulfilment of the supporting features, and the patient showed good visual recovery by oral glucocorticoid therapy. The patient presented with new-onset bilateral numbness and bladder and bowel dysfunction. Thoracic MRI revealed a short, non-central lesion at the T8 level, which did not fulfil the supporting features for myelitis (Fig. 2E).

Case 4: An adult female with a long-standing history of demyelinating events, including ON and recurrent myelitis. She initially developed right ON several decades earlier, followed by multiple episodes of short thoracic myelitis, for which the supporting features could not be assessed. The most recent cervical lesion at the C1 level did not fulfil the supporting features (Fig. 2F). Serum MOG-IgG was low positive (1:64), while CSF was negative. Two investigators judged that these episodes did not fulfil the supporting features. In both Cases 3 and 4, MS was considered because of the short, peripheral spinal cord lesions and history of ON. However, CSF oligoclonal bands and IgG index were negative in both cases, and brain MRI showed no lesions suggestive of multiple sclerosis. Both Cases 3 and 4 have remained relapse-free for more than 7 years: Case 3 without maintenance treatment and Case 4 on low-dose oral prednisolone (10 mg/day). Therefore, making a diagnosis of MS in these cases was considered difficult.

Among these four cases that did not fulfil any supporting features, one case met the MOGAD panel criteria due to clear positivity, whereas the remaining three cases, with either low-positive serum or CSF-restricted MOG-IgG, did not (Table 2).

### Correlations between fulfilled supporting features and MOG-IgG titres in sera and CSF

The correlation coefficients between the numbers of the fulfilled supporting features and MOG-IgG titres in serum and CSF were as follows: *r* = 0.11 (adjusted *P* > 0.9) for serum titres and *r* = 0.16 (adjusted *P* > 0.9) for CSF titres in ON, *r* = 0.31 (adjusted *P* = 0.11) for serum titres and *r* = 0.08 (adjusted *P* > 0.9) for CSF titres in myelitis, and *r* = 0.39 (adjusted *P* = 0.0053) for serum titres and *r* = 0.26 (adjusted *P* = 0.27) for CSF titres in cerebral and brainstem/cerebellar syndrome. In the supporting features of cerebral and brainstem/cerebellar syndrome, MOG-IgG titres in serum were significantly higher in the cases with multiple ill-defined T2 hyperintense lesions than those without such lesions (adjusted *P* < 0.001). However, MOG-IgG titres in serum did not differ in the other three supporting features in cerebral and brainstem/cerebellar deficits (adjusted *P* = 0.14 for deep grey matter lesion, adjusted *P* > 0.9 for lesions in pons, middle cerebellar peduncle, or medulla, and adjusted *P* = 0.80 for cerebral cortical lesions).

### Frequency of fulfilled supporting features linked with MOG-IgG titres in sera and CSF

The frequencies of meeting the supporting features showed a titre-dependent trend: in serum, 92% (12/13) in seronegative/CSF-restricted cases, 88% (15/17) at 1:16–1:64, 97% (38/39) at 1:128–1:512, and 100% (72/72) at ≥1:1024; in CSF, 92% (46/50) at negative–1:4, 100% (66/66) at 1:8–1:32, and 100% (25/25) at ≥1:64 (Table 3: Relationship between supporting features and MOG-IgG titres in serum and/or CSF). When assessing serum-CSF combinations, fulfilment of the supporting features was lower in cases with CSF titres of negative ∼1:4, including 2 of 3 (67%) with negative serum, 7 of 9 (78%) with serum titres of 1:16–1:64, and 20 of 21 (95%) with serum titres of 1:128–1:512. In all other combinations, all cases fulfilled the supporting features.

**Table 3:** The relationship between MOG-IgG titres and supporting features

MOG-IgG-serum-titre	MOG-IgG-CSF-titre	Cases fulfilling at least one of the supporting features, *n*/*N* (%)
Seronegative (= CSF restricted)		12/13 (92%)
1:16–1:64		15/17 (88%)
1:128–1:512		38/39 (97%)
≥1:1024		72/72 (100%)
	CSF-negative (=serum-restricted MOG-IgG)-1:4	46/50 (92%)
	1:8–1:32	66/66 (100%)
	≥1:64	25/25 (100%)
Negative	Negative-1:4	2/3 (67%)
Negative	1:8–1:32	9/9 (100%)
Negative	≥1:64	1/1 (100%)
1:16–1:64	Negative-1:4	7/9 (78%)
1:16–1:64	1:8–1:32	7/7 (100%)
1:16–1:64	≥1:64	1/1 (100%)
1:128–1:512	Negative-1:4	20/21 (95%)
1:128–1:512	1:8–1:32	10/10 (100%)
1:128–1:512	≥1:64	8/8 (100%)
≥1:1024	Negative-1:4	17/17 (100%)
≥1:1024	1:8–1:32	40/40 (100%)
≥1:1024	≥1:64	15/15 (100%)

The proportions of cases fulfilling at least one of the supporting features stratified by the titres of MOG-IgG in serum and CSF. A titre of 1:128 in serum was set as the cut-off value for clear positivity in this study.

### MOG-IgG-positive cases that did not meet the current criteria

Consequently, a total of five cases (3.5%) did not meet the MOGAD panel criteria: two (1.4%) showing clear positivity of MOG-IgG in serum, yet lacking the core events, and three (2.1%) meeting the core events but displaying no supporting features and low positivity in serum or CSF-restricted MOG-IgG (Fig. 3: Sankey diagram showing performance of the MOGAD panel criteria).

**Figure 3:**
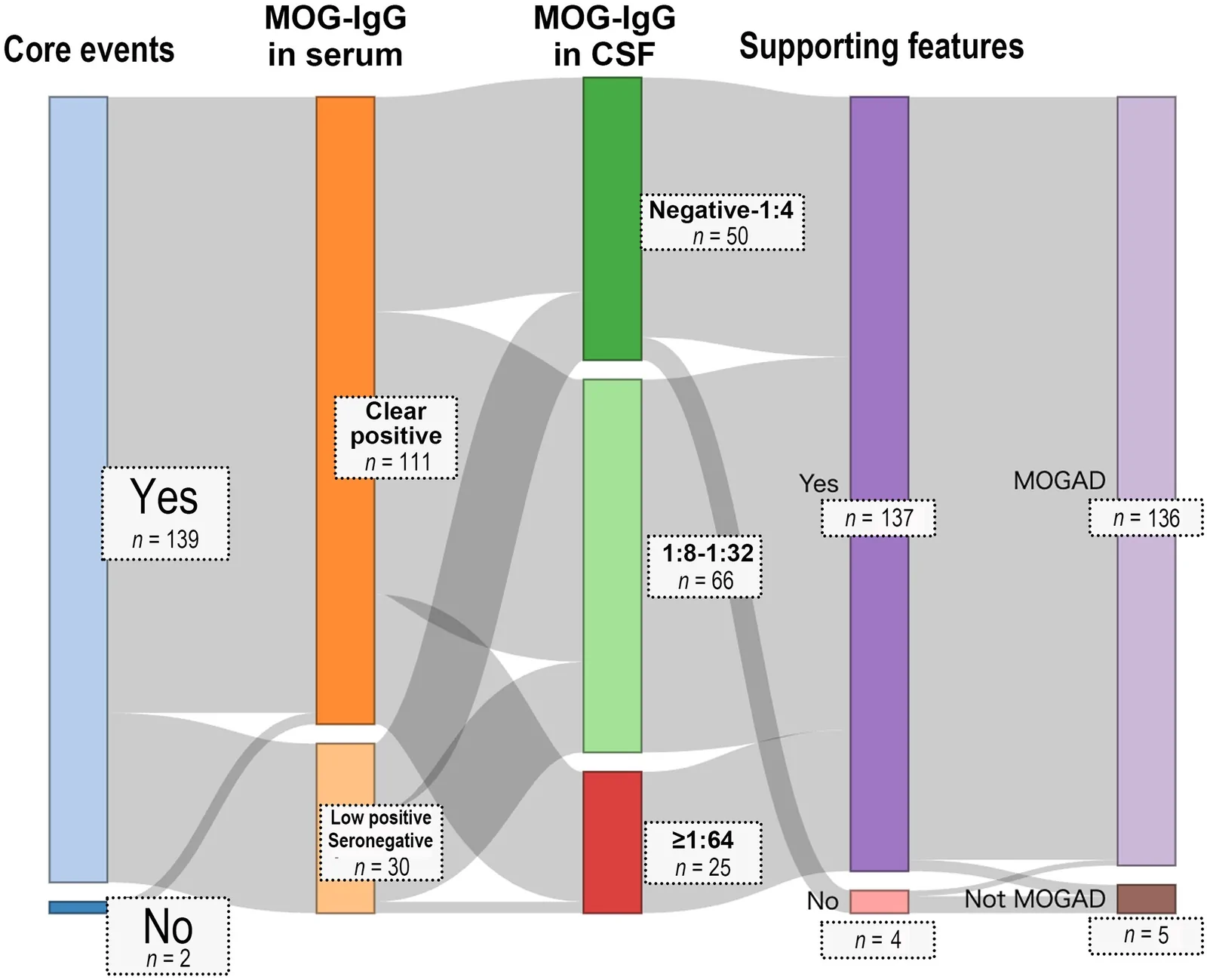
**Sankey diagram showing performance of the MOGAD panel criteria.** Classification of cases based on the core events, MOG-IgG status in serum and CSF, and fulfilment of the supporting features. Of the 141 cases, 139 fulfilled the core events, while two meningitis cases did not. Serum MOG-IgG was clearly positive in 111 cases, while 30 were low positive or seronegative (CSF-restricted MOG-IgG). In CSF, 50 cases were negative or had titres up to 1:4, 66 had titres of 1:8–1:32, and 25 had titres ≥1:64. Supporting features were fulfilled in 137 cases and absent in four. Overall, 136 cases fulfilled the MOGAD panel criteria, while five did not. IgG, immunoglobulin G; MOG, myelin oligodendrocyte glycoprotein; MOGAD, myelin oligodendrocyte glycoprotein antibody-associated disease.

## Discussion

This study clarified the following aspects in our cohort of CNS demyelinating disease with MOG-IgG positivity in serum and/or CSF: (1) the proportion of cases meeting the core events or supporting features, (2) the characteristics of patients not meeting them and (3) the association between the supporting features and MOG-IgG titres in serum and CSF.

In the present study, five cases (3.5%) did not fulfil the MOGAD panel criteria. Among them, two cases of aseptic meningitis with clear positive findings did not fulfil any core events; however, they had such supporting features as multiple ill-defined T2-hyperintense lesions and/or deep grey matter lesions. These two cases had been classified as MOGAD based on ADEM-like lesions in our previous study.^12,17^ According to several previous studies, aseptic meningitis, prolonged fever, and isolated intracranial hypertension have been reported as distinct features or atypical presentations of MOGAD (Table 4).^18-39^ However, these phenotypes are not mutually exclusive; they may overlap and evolve into one another over time. For example, patients with aseptic meningitis may present with preceding prolonged fever and elevated intracranial pressure.^19-21^ These conditions frequently progress to ON, myelitis, ADEM, or cortical encephalitis, eventually fulfilling the MOGAD panel criteria. Notably, 16 of 21 previously reported cases of aseptic meningitis, prolonged fever or intracranial hypertension later developed ON, myelitis, ADEM, brainstem/cerebellar deficits or cortical encephalitis and ultimately met the diagnostic criteria. However, some remained as isolated presentations with only prolonged fever and/or intracranial hypertension, like our cases, which did not meet the present criteria.^24,25^ Therefore, these features should not only be viewed as prodromes of MOGAD but may need to be considered as distinct core events within the MOGAD spectrum. In such cases, clear positivity of MOG-IgG might be required for MOGAD diagnosis, as suggested by the present study. Recent evidence by Aboseif *et al*. similarly reported that CSF MOG-IgG was positive in all tested cases with meningitis, as was serum MOG-IgG (5/5).^28^ This suggests that CSF MOG-IgG may serve as a useful diagnostic marker in patients presenting with a meningitis-like syndrome.

**Table 4:** Aseptic meningitis, prolonged fever, and intracranial hypertension reported as atypical presentations of MOGAD in the literature

Publication	Number of cases	Age	Sex Ratio (M: F)	Symptoms/Findings	2023 IPMOGAD criteria fulfilled?
Lotan *et al*., 2018^18^	5	6–50	2: 3	Symptoms: headache, blurry vision. **OP: 23–35 cmH_2_O**. MRI: optic nerve enhancement (100%).	**Yes**
Suzuki *et al*., 2019^29^	1	55	F	Symptoms: headache, nausea, vomiting, and fever, later developed left ON. OP: 25 cmH_2_O. MRI: superficial cortical hyperintensity, later left ON.	**Yes**
Narayan *et al*., 2019^19^	1	18	F	Symptoms: blurred vision, headache, photophobia. **OP: 29 cmH_2_O.** MRI: optic nerve enhancement, cervical and short thoracic cord lesions.	**Yes**
Valdrighi *et al*., 2021^20^	1	12	F	Symptoms: headache and double vision. **OP: 52 cmH_2_O**. MRI: multifocal leptomeningeal enhancement and pontine lesions.	**Yes**
Udani *et al*., 2021^21^	12	3–11	8: 4	Symptoms: Fever **(100%)**, headache (50%), vomiting (42%), and seizure (25%). OP: ND. MRI: ADEM (50%), ON (25%), TM (8%), brainstem lesions (8%), white matter and brainstem lesions (8%).	Not assessable
Lampros *et al*., 2021^22^	1	25	M	Symptoms: fever and headache. OP: ND. MRI: LETM, corpus callosum and brainstem lesions.	**Yes**
Jeantin *et al*., 2022^23^	1	35	F	Symptoms: headache, nausea, abdominal pain, acute onset urinary retention. **OP: 31 cmH_2_O**. MRI: short cervical lesion. MOG-IgG: Serum and CSF positive.	**Yes**
Chaudhuri *et al*., 2022^24^	1	19	F	Symptoms: blurred vision, severe headache, vomiting. OP: 25–35 cmH_2_O. MRI: a**bnormal unilateral leptomeningeal enhancement**. MOG-IgG positive in both serum (ND) and CSF (1:10).	**No**
Lin *et al*., 2022^25^	2	5, 10	2:0	Symptoms: fever, headache, and vomiting, and persistent headache. OP: 26–30 cm H_2_O. No focal neurological manifestations. MRI: (Case 1) initially normal, later developed multiple brain lesions. (Case 2) normal.	No
Wong *et al*., 2022^30^	3	4–12	0: 3	Symptoms: headache (66%), seizure (66%), and fever (66%). **OP: >40, >32, 60 cmH_2_O**. MRI: multifocal brain lesions (100%). MOG-IgG: seropositive (titres were not specified)	Yes
Azar *et al*., 2023^26^	1	24	F	Symptoms: fever, brachiofacial paresthesia, phonophobia, photophobia, and urinary retention. MOG-IgG seropositive (1:160).	Yes
Alqahtani *et al*., 2023^31^	1	12	M	Symptoms: prolonged severe headache. OP: 35 cmH2O. MOG-IgG seropositive (1:320).	No
Chakraborty *et al*., 2023^32^	1	21	F	Symptoms: decreased visual acuity bilaterally. OP: 22 cm H2O. MOG-IgG: seropositive.	Yes
Zhou *et al*., 2023^33^	1	12	M	Symptoms: headache, later developed ADEM. **OP >36 cmH_2_O**. MRI: multifocal cortical/subcortical T2 hyperintensities. MOG-IgG seropositive (1:40; low positive)	Yes
Maran *et al*., 2023^34^	1	10	M	Symptoms: nausea, vomiting, epistaxis and a sharp bitemporal headache, later developed unilateral ON. **OP: 65 cmH_2_O**. MRI: initially normal, but later multiple subcortical white matter lesions were observed. MOG-IgG: seropositive (1:320).	Yes
Gu *et al*., 2023^27^	16	7–52	9: 7	**Symptoms: fever (87.5%)**, headache (75%). OP: >20 cm H_2_O in 43.8%, >30 cm H_2_O in 18.8%. MOG-IgG: all seropositive, 81.3% of patients were positive in both serum and CSF.	Not assessable
Nguyen *et al*., 2024^35^	9	ND	3: 6	**Clinical phenotypes: 7 ADEM, 1 ON, 1 Other. OP: > 28 cm H2O.**	**Yes**
Sorensen *et al*., 2024^36^	1	53	F	**OP: markedly elevated.** Symptoms: headache, decreased vision. MRI: initially normal, but repeat orbital MRI showed symmetric thin optic nerve sheath. MOG-IgG: seropositive (1:100).	No
Papetti *et al*., 2024^37^	1	10	M	Symptoms: headache, vomiting, later developed ON. **OP: 75 cmH_2_O**. MRI: no brain or spinal cord abnormalities suggestive of MOGAD. MOG-IgG: both serum and CSF were positive (1:100).	**Yes**
Nagaratnam *et al*., 2024^38^	2	21, 44	1: 1	Symptoms: fever (100%), headache (100%), decreased consciousness (50%) and vertigo (50%). **OP: 35, 43 cm H_2_O**. MRI: both had normal initial imaging, with subsequent multiple brain lesions. MOG-IgG: Case 1 had serum and CSF MOG-IgG. Case 2 had CSF-restricted MOG-IgG.	Yes
Han *et al*., 2025^39^	3	3–9	0: 3	Symptoms: fever (66%), seizure (66%), myelopathy (33%). OP: ND. MRI: leptomeningeal enhancement (66%).	Yes
Aboseif *et al*., 2025^28^	34	4–61	21: 13	Symptoms: headache (94%), fever (62%). OP: elevated in 11/18 cases (61%). MRI: leptomeningeal abnormality in 7/34 cases (21%). MOG-IgG: all seropositive; CSF-positive in all 5 tested cases.	Not assessable a

ADEM, acute disseminated encephalomyelitis; F, female; LETM, longitudinally extensive transverse myelitis; M, male; ND, not described; ON, optic neuritis; OP, opening pressure. For cases not explicitly classified according to the IPMOGAD 2023 criteria in the original reports, we assessed their fulfilment based on the available clinical and imaging information.

^a^17 cases (50%) evolved into a core clinical demyelinating event.

The manifestations of two cases in our cohort that did not fulfil any supporting features were ON or myelitis. Kim *et al*. reported that 93 of 100 cases of MOGAD, including 36 with clear MOG-IgG positivity, met the criteria.^2^ As in our study, the remaining seven cases that did not meet the criteria all presented with ON (*n* = 3) or myelitis (*n* = 4) in the Korean cohort (Table 5. Summary of the studies on the validation of the MOGAD panel criteria).^2-8,40-45^ Not all reported MOGAD cases fulfil the supporting features. In ON: 31–58% with bilateral involvement,^46-50^ 80% with longitudinal involvement,^49^ 50% with optic sheath enhancement,^49^ 76–95% with papilledema.^48,49,51^ In myelitis: more than 60% with longitudinal involvement,^52-55^ 30–75% with central cord lesion or H-sign,^52,53,56^ 26% with conus involvement. In cerebral and brainstem/cerebellar syndrome: ADEM in 50% of children, 5.6% of adults. Deep grey matter involvement 13–50%, 60–83% with brainstem or cerebellar involvement, 14.7% with cerebral cortical lesions.^57^ Therefore, systematic clinical and MRI assessment is crucial when applying the 2023 MOGAD panel criteria. In particular, axial spinal cord T2-weighted images are important not only for identifying MOGAD-supportive features, such as central cord lesions or H-sign, but also for detecting short peripheral cord lesions suggestive of MS. Thus, systematic axial spinal cord imaging is important for both application of the 2023 MOGAD panel criteria and exclusion of alternative diagnoses.

**Table 5:** Summary of the research on the validation of IPMOGAD criteria (2023)

Authors (year of publication, country)	MOG-IgG positive cases, *n*	CSF MOG-IgG-positive cases, *n*	The frequency of fulfilling supporting features in clear-positive cases, *n*/*N* (%)	The frequency of fulfilling supporting features in low-positive cases, *n*/*N* (%)	The frequency of fulfilling the IPMOGAD criteria (2023) in serum- and/or CSF-positive cases, *n*/*N* (%)	The frequency of fulfilling the IPMOGAD criteria (2023) in CSF-positive cases, *n*/*N* (%)	The frequency of fulfilling the IPMOGAD criteria (2023) in CSF-restricted-positive cases, *n*/*N* (%)
Kim *et al.* (2023, Korea)^2^	100	NR	35/35 (100%)	57/64 (89%)	93/100 (93%)	NR	NR
Forcadela *et al.* (2024, UK)^3^	127	2	103/103 (100%)	21/24 (88%)	123/127 (97%)	2/2 (100%)	1/1 (100%)
Alaboudi *et al.* (2024, US)^5^	27	NR	NR	11/16 (69%)	18/27 (67%)	NR	NR
Kurd *et al.* (2024, Israel)^7^	28	NR	NR	NR	28/28 (100%)	NR	NR
Risi *et al.* (2024, Italy)^8^	38	NR	NR	8/8 (100%)	38/38 (100%)	NR	NR
Varley *et al.* (2024, UK)^6^	103	NR	NR	NR	86/103 (84%)	NR	NR
Filippatou *et al.* (2024, US)^4^	122	NR	63/64 (98%)	46/58 (79%)	109/122 (89%)	NR	NR
Rechtman *et al.* (2024, Israel)^40^	88	NR	NR	NR	86/88 (98%)	NR	NR
Fonseca *et al.* (2024, Spain)^41^	257	8	150/158 (95%)	31/36 (86%)	240/257 (93%)	4/8 (50%)	4/8 (50%)
Contentti *et al.* (2024, Latin America)^45^	171	NR	19/19 (100%)	38/48 (79%)	144/171 (84%)	NR	NR
Cai *et al.* (2025, China)^42^	291	NR	NR	195/197 (99%)	262/291 (90%)	NR	3/4 (75%)
Kwon *et al.* (2025, Korea)^44^	93	NR	43/43 (100%)	23/26 (88%)	66/93 (71%)	NR	NR
Carta *et al.* (2025, Italy)^43^	214	41	65/89 (73%)	90/125 (72%)	154/184 (84%)	NR	8/9 (89%)
Present study (2026, Japan)	141	116	110/111 (99%)	15/17 (88%)	136/141 (96.5%)	113/116 (97%)	12/13 (92%)

NR, not reported.

Thus, future studies are warranted to clarify the characteristics of cases that did not meet any supporting features and to identify biomarkers that may increase the sensitivity of the criteria, such as advanced MRI techniques, optical coherence tomography, and cytokine analyses.^17,58-62^ In addition, one case experienced a relapse with ON and did not fulfil any supporting features at the time of MOG-IgG testing, but had previously fulfilled the supporting features during an earlier episode of ADEM in this study. Considering prior episodes that met one or more supporting features, rather than relying solely on findings at the time of MOG-IgG testing, may increase the sensitivity of the diagnostic criteria without compromising specificity in some cases.

In the 2023 MOGAD panel criteria, clear positivity of serum MOG-IgG is sufficient for diagnosis in patients with a core clinical demyelinating event after exclusion of alternative diagnoses.^1^ In contrast, CSF-restricted MOG-IgG is treated similarly to low-positive serum MOG-IgG, requiring one or more supporting features. In this study, cases with higher CSF MOG-IgG titres fulfilled supporting features at a frequency similar to cases with clear-positive serum MOG-IgG, even when serum MOG-IgG was low positive. Our previous study also showed that a higher CSF MOG-IgG cut-off (≥1:8) in our in-house live CBA effectively excluded typical MS.^12^ These findings suggest that high-titre CSF MOG-IgG may have diagnostic reliability comparable to clear-positive serum MOG-IgG and should be considered as a potential equivalent in future revisions of the MOGAD panel criteria, particularly when only CSF is tested in clinical practice. For live CBAs detecting MOG-IgG in CSF, defining clear positivity as at least two doubling dilutions above the assay cut-off, analogous to serum testing, may be a practical approach.^1^

Higher MOG-IgG titres in serum were associated with multiple ill-defined T2 hyperintense lesions. Perivenous demyelination was initially reported in ADEM and then found to be a characteristic pathological finding of MOGAD.^63-65^ We previously reported that the ADEM phenotype was more frequent in the group with higher serum MOG-IgG titres than in those with lower titres,^12^ although the precise mechanism underlying the effect of serum titres on multiple brain lesions is unknown.

The present study has several limitations. First, our cohort primarily comprised individuals of Japanese ethnicity, necessitating validation in other ethnic groups. Second, because this was a retrospective, cross-sectional study, some supporting features were not fully assessable, future relapses that might fulfil the criteria were not captured, and previous attacks were not used as qualifying events in the primary assessment. In particular, ON-related supporting features may have been underestimated because funduscopic assessment was not standardized across centres and OCT data were not systematically collected. Third, selection bias cannot be completely excluded, as the primary analysis was restricted to patients with paired serum and CSF samples (For example, CSF examinations might be performed less frequently in patients with ON than in those with encephalitis or myelitis). Although we additionally reviewed patients for whom only serum (1302/3904, 33%) or only CSF (*n* = 21) was available and found a comparable distribution of major clinical phenotypes, including ON, myelitis and ADEM, this does not necessarily indicate that the frequency of supporting features or atypical features was also comparable. Finally, CSF MOG-IgG testing was performed in a single laboratory using an in-house live cell-based assay and has not yet been validated in multicentre studies. Because assay performance may vary depending on laboratory protocols, secondary antibodies, and cut-off definitions, our CSF findings should not be directly generalized to other laboratories without further validation.

## Conclusions

The performance of the 2023 MOGAD panel criteria was validated in a Japanese cohort in which both serum and CSF were systematically tested. Overall, the criteria demonstrated good performance; however, a small number of cases with meningitis, prolonged fever, ON, or myelitis did not fulfil the MOGAD panel criteria. Higher titres of MOG-IgG in both serum and CSF were associated with a higher likelihood of fulfilling one or more supporting features. High-titre CSF MOG-IgG may be diagnostically reliable and should be considered as a potential equivalent to clear-positive serum MOG-IgG in future revisions of the criteria, although multicentre validation of CSF testing is required. Future revisions of the criteria may need to incorporate less common presentations and further evaluate the diagnostic significance of CSF-restricted or low-positive MOG-IgG findings after careful exclusion of alternative diagnoses.

## Data Availability

The datasets that support the findings of the current study are available from the corresponding author upon reasonable request. The data are not publicly available because they contain information that could compromise the privacy of the study participants.
